# Immune system ontogeny—a subtle evolution to perfection

**DOI:** 10.3389/fragi.2026.1872955

**Published:** 2026-09-29

**Authors:** Adriana Narcisa Munteanu, Mihaela Surcel, Elena Georgiana Dobre, Carolina Constantin, Monica Neagu

**Affiliations:** 1 Immunology Laboratory, “Victor Babes” National Institute of Pathology, Bucharest, Romania; 2 Pathology Department, Colentina Clinical Hospital, Bucharest, Romania; 3 Doctoral School of Biology, Faculty of Biology, University of Bucharest, Bucharest, Romania

**Keywords:** ageing, immunity, immunological memory, immunosenescence, inflammaging, ontogeny

## Abstract

It is well known that both innate and adaptive immune systems in newborns are immature, making them highly susceptible to infection. Additionally, pre-term newborns with hindered immune systems are more prone to increased mortality and morbidity. Newborns are capable of mounting adaptive immune responses, but their capability to develop long-lasting immunity is limited. Thus, the fetus and newborn’s immune system is considered “immature” due to functional disorders in immune response or suppression by regulatory cells. Aging is another factor that leads to impaired immune responses due to a state of chronic low-grade inflammation (inflammaging), which compromises defense against pathogens and malignancies and favors the onset of age-related disorders. However, in this complicated scenario, centenarians emerged as a paradigm shift due to their particular immune system that prevents inflammaging and enables them to achieve longevity. Therefore, the present review aims to provide an updated overview of immune response development through neonatal, infant, and adult life in order to highlight stage-specific mechanisms and uniquely regulated immune responses. Taken together, this information provides mechanistic insights that can be harnessed to develop age-adapted interventional strategies and prevent the onset of age-associated disorders in older adults.

## 1 Introduction

In the course of evolution, humans have developed specific immunity mechanisms and immunological memory to effectively respond to acute and chronic infections, which has ensured their survival as species. The human immune system continuously evolves throughout life. It is a mirror of each individual’s lifetime history, where geographical environment, nutrition, and lifestyle shape the genetically-driven immune architecture. In general, at birth, both innate and adaptive immunity are relatively immature, relying on maternal antibodies (Abs) for early protection (Esser et al., 2026). The child’s immune system thus exhibits a degree of immaturity that persists from birth to around 7 years of age. Immaturity may be due to functional disorders in the immune response or suppression by regulatory cells, so that the child’s immune response is considered to be “hypo-inflammatory”. Throughout this period, the normal child can contract a series of infections, mostly subclinical, that contribute to strengthening their immune system, expanding immunological memory, and increasing the efficiency of the immune response—conditions that will contribute to the maturation of the immune system in the prepubertal period (Munteanu et al., 2019). If significant clinical infections are repeated and prolonged, the evolution of the disease or various complications (pulmonary, cutaneous, etc.) can occur during childhood.

Starting from childhood, immune responses became mature, memory is established, and the system then reaches full efficiency in adulthood. In women during pregnancy, the physiological parameters of the immune system will change toward a tolerogenic status, and the immune system will begin to decrease in potency in all individuals as they age. In elderly adults, immunosenescence and chronic inflammation may lead to a gradual decline in immune function, increasing susceptibility to infections, autoimmune diseases, and chronic inflammation (Munteanu et al., 2022). However, this does not apply to all individuals: there is also a model of “healthy” aging experienced by centenarians, who not only experience a longer lifespan and greater independence but also exhibit reduced levels of chronic inflammation, a lower prevalence of age-related disorders, and remarkable pathogen clearance and increased self-tolerance (Zhang et al., 2026). Centenarians are a population that shows increased adherence to healthy lifestyles, often characterized by a balanced diet, physical activity, reduced stress, strong social involvement, and reasonable sleep (García-Vivanco et al., 2025). Thus, the evolution of the human immune system reflects a progressive refinement from simple innate defenses to sophisticated adaptive mechanisms, influenced by genetic innovation, environmental challenges, lifestyle factors, and pathogen pressures. Understanding this evolutionary history may provide valuable information on human health, disease susceptibility, and the development of age-related vaccines and immunotherapies.

The advancement of high-throughput omics technologies, such as single-cell RNA-sequencing (scRNA-seq), has enabled the analysis of the human immune system at unprecedented resolution, generating comprehensive atlases that have revealed dynamic, age-related changes in immune cell subsets (Nehar-Belaid et al., 2026). For instance, scRNA-seq analysis of three centenarian cohorts revealed an increase in peripheral natural killer (NK) cells frequencies alongside a reduction of B and T-CD4^+^ cell counts in centenarians compared with control subjects (Wang B. et al., 2025). In parallel, Zhang et al. (2026) developed a bioinformatic algorithm to identify the peripheral immune cell populations that positively and negatively associate with longevity. Interestingly, cytotoxic immune cells such as NK, T-CD8^+^, and γδ T cells positively correlate with longevity, whereas cellular populations involved in inflammatory pathways such as T-CD4^+^, B, and dendritic (DCs) cells display negative associations with extended lifespans. Thus, it seems that the maintenance of cytotoxic immune phenotypes and the attenuation of inflammatory responses is a feature of healthy aging that may be exploited to mitigate immunosenescence in clinical settings (Zhang et al., 2026). In parallel, Nehar-Belaid et al. (2026) have highlighted the immunological status of infants as completely differing from other age groups. In brief, they showed that infants, who are the most vulnerable to respiratory infections due to their immature immune system, compensate for these limitations by harboring elevated frequencies of peripheral pDC and SOX4^+^ naïve T cells and considerable amounts of interferon (IFN)-γ released from T-CD4^+^ cells. Taken together, all these data highlight potential new mechanisms that may be exploited to sustain infant immunity and restore immune function in older, frailer individuals.

Our review also focuses on the concept of immunological memory and how aging impacts its proper functioning. Immunological memory is the ability of the adaptive immune system to respond efficiently to a second encounter with a pathogen and is the basis of vaccination. Immunological memory generated during youth persists into late adulthood, whereas that generated in the elderly is not as effective as they are less capable of responding to vaccines. Nevertheless, memory-like responses have also been reported in innate immune cells and have led to the concept of “trained immunity”, which involves a significant epigenetic and metabolic reprogramming of hematopoietic progenitors to ensure enhanced protection against secondary unrelated infections during lifetime—of particular importance in vulnerable groups. Here, we will emphasize key findings related to immunological memory and trained immunity in different age groups, highlighting the challenges and benefits associated with exposure to immunostimulants in early life, but also the different perspectives that may arise from immunological memory evaluation in adults and the elderly.

The present review will elaborate on details concerning the immune elements that evolve during ontogeny and how our immune system surveys the constant challenges and aggressor attacks during our lifespan. Emphasis is placed on the associations between the dynamic shifts in immune cell populations and the biological effects observed at specific developmental stages to better understand the vulnerabilities and opportunities that may arise during each developmental window.

## 2 The innate immune system

The nonspecific (innate) immune system acts as a first line of defense against pathogens, and nonspecific immune mechanisms are present from birth to the end of life, manifesting the same intensity on all antigens (Ags). The nonspecific system ensures a fully activated non-specific response within minutes to hours of interaction with the viral/bacterial pathogenic agent.

Natural resistance is ensured by passive tissue barriers and factors which constitute the basic defense against the environment, and by active factors (humoral and cellular) that actively neutralize aggressors. When an infectious agent overcomes the natural barriers (skin, mucous membranes, and endothelia), non-specific fluids such as urine and tears serve to carry microbes away from the normally sterile environment of the urinary tract or eyes (Mudgil, 2022; Reasoner et al., 2025). Another fluid, gastric acid, creates a highly acidic environment, lethal to many ingested microorganisms, that neutralizes potential threats before they can proceed to the intestines (Traulsen et al., 2021). The pulmonary system has a mucociliary clearance mechanism that synthesizes mucus enriched with chemical barriers such as antimicrobial proteins and immunoglobulin (Ig) A (Kageyama et al., 2024). If these barriers are surpassed, various immune innate actors come into play, such as cells and humoral secreted factors that actively neutralize pathogens. Once innate immune cells such as monocytes (Mo), macrophages (Mf), neutrophils (Neu), and DCs are activated, a myriad of factors are secreted to neutralize the agent. These include factors such as interferons and complement system molecules which work in synergy with cellular processes such as phagocytosis, pinocytosis, and direct cytotoxicity to counteract infectious agents. Innate lymphoid cells (ILCs), including NK, NKT, and γδT cells, are also part of this innate immune army; their particular contributions will be further discussed below.

### 2.1 Macrophages

Mf and their precursors, the circulating Mo, are elite guardians against various pathogenic and environmental insults, playing key roles in phagocytosis, inflammation, wound healing, and tissue homeostasis. Mf reside in tissues, comprise 10% of immune cells in the human body, and are subdivided into two major phenotypes: M1-Mf, with antimicrobial, proinflammatory properties, and immunosuppressive M2-Mf, based on their similarities with the T helper (Th)1/Th2 cell subtypes (Yao et al., 2019). The maintenance of the M1/M2 balance is essential for maternal-fetal tolerance and for a proper gestational evolution—a reduction in M2-Mf numbers has been reported in the decidual tissues of preterm preeclamptic pregnancies compared to preterm control pregnancies (Schonkeren et al., 2011). Furthermore, analysis of cells from the bronchoalveolar lavage of term and preterm infants have revealed an increased abundance of non-classical (CD14lowCD16^+^) Mo in preterm infants on the day of delivery, suggesting that gestational age may dictate Mf phenotype and infant susceptibility to respiratory disorders (Prince et al., 2014). In newborns, Mo and classical Mf are still immature cells that exhibit diminished signaling pathways and responses to cytokines compared to the adult population, but normal levels are reached a few days after birth. Within this process, the microbiota of the newborn and the colonization mechanism of intestinal flora in their early life are the first “teachers” of a developing immune system (Ma et al., 2023). Ageing has a significant impact on Mo/Mf phenotype and functions, resulting in reduced phagocytosis and autophagy concurrent with Toll-like receptor (TLR) downregulation, the upregulation of senescence markers, and the elevated production of inflammatory cytokines (De Maeyer and Chambers, 2021). Interestingly, Komaravolu et al. (2026) have highlighted that age-induced changes in chemokine receptor expression shape specific Mo phenotypes associated with inflammaging. However, they reported a decrease in classical Mo (CD14^+^CD16^−^) with an increase in immature Mo with aging. Additionally, another study reported higher CD40 expression on classical and non-classical Mo in frail, older individuals compared with healthy older subjects, without any differences in Mo subset distributions among groups, thus highlighting a potential correlation between Mo-CD40^+^ and aggravated, frail conditions (Reitsema et al., 2024).

### 2.2 Neutrophils

Neu are specialized microbial hunters due to their migratory, degranulating, cytotoxic, and phagocytic capabilities. Mature Neu cells are already present in the fetal circulation at the end of the first trimester of pregnancy; their number increases sharply before birth and stabilizes within a few days. From the initial appearance of Neu in the liver, thymus, and spleen (at around the eighth week of human gestation) to their generation in large numbers in the bone marrow at the end of term gestation, there is a complex process of differentiation. However, the differentiation of pluripotent hematopoietic stem cells into mature, segmented Neu is a highly controlled process where the transcriptional regulators CCAAT/enhancer-binding protein (C/EBP)-α and C/EBP-ε play a vital role. Recent advances in Neu biology have clarified the life cycle of these cells and revealed striking differences between neonatal and adult neutrophils based on fetal maturation and environmental factors (Lawrence et al., 2018). The establishment of a healthy neonatal microbiome after birth is vital for maintaining Neu homeostasis and proper cellular functioning. The coevolution of mammals and their microbiota have led to a reliance on microbiota-derived signals to provide tonic stimulation to the systemic innate immune system, further maintaining immune vigilance against infection (Balmer et al., 2014). Host microorganisms induce the production of interleukin (IL)-17 by group 3 ILC (ILC3) in the intestine, thereby increasing granulocyte colony-stimulating factor (G-CSF) and, hence, Neu production in a TLR-4- and myeloid differentiation primary response (Myd)88-dependent manner (Deshmukh et al., 2014). Neu have a weak inflammatory response, and their deficiency is more obvious in premature infants, who are more exposed to bacterial infections (Filias et al., 2011). Despite numerous studies, it is still unclear whether counts of Neu increase or remain constant over a lifetime (Valiathan et al., 2016; Tang et al., 2024). However, what is certain is that Neu functions may be significantly impacted by aging; for example, increased apoptotic rates have been reported in Neu from elderly individuals, leading to a decrease in their antimicrobial roles due to the impairment of phagocytosis and NET formation (Clark et al., 2021; Wang Z. et al., 2025). A noteworthy paradox is that of centenarians, who represent a model of healthy aging in terms of both lifespan and the degree of independence maintained. A comparison of Neu functions and antioxidant parameters in peripheral blood across three age-stratified cohorts revealed that frail, middle-aged subjects exhibit Neu with lower phagocytic and chemotactic activities and increased superoxide anion levels compared to young adults and centenarians; the latter two groups displayed comparable Neu functions and glutathione levels (Alonso-Fernández et al., 2008). This aligns with other studies that have highlighted lower superoxide anion levels in centenarian Neu, as well as reduced levels of oxidized proteins and lipids in centenarian tissues compared to middle-aged groups. This underscores redox balance as an essential condition for maintaining Neu in a fully functional state independent of age (Belenguer-Varea et al., 2020).

### 2.3 Dendritic cells

DCs are immune sentinels that bridge innate and adaptive responses due to their ability to migrate in draining lymph nodes and initiate Ag-specific immune responses. Functionally, DCs may be subdivided into conventional/classical DCs (cDCs) and plasmacytoid DC (pDC). While cDCs are the main activators of adaptive immune responses due to their ability to process and present Ags to T cells, type I-IFN-producing pDC constitute the immune system’s defense against viral infections (Papaioannou et al., 2019). Based on their transcription factor dependence and surface markers, cDCs have been subdivided into two groups: 1) cDC1 cells, which express interferon regulatory factor (IRF)8 and basic leucine zipper ATF-like transcription factor (BATF)3, activate T-CD8^+^ cells via cross-presentation, and orchestrate Th1 cell responses against intracellular pathogens; 2) cDC2, the main task of which is to induce Th2 and Th17 responses towards extracellular pathogens (Muleta et al., 2026). Myeloid-like DCs (mDCs) are present in small numbers in the umbilical cord and secrete IL-12p70 at low concentrations. IL-12p70 plays an important role in the induction of NK cells and in the activation, differentiation, and proliferation of specific Th1 lymphocytes that specialize in eradicating pathogenic bacteria. The responses of Th1 and T-CD8^+^ lymphocytes are diminished, so that the susceptibility to infections caused by viruses and bacteria is increased. In contrast, mDCs in newborns stimulated by the transmembrane protein TLR4 (receptors with an important role in pathogen recognition and activation of the nonspecific immune response) induce the production of inflammatory cytokines involved in the activation of nonspecific immune system in concentrations similar to adults (Willems et al., 2009).

Interestingly, Nehar-Belaid et al. (2026) have highlighted that the proportion of DCs is lowest in infants than in other age groups (e.g., children, adolescents, young adults, middle-aged adults, and the elderly), with pDCs constituting 53% of the total DC population. Previous studies reported similar absolute pDCs counts in term and preterm newborns and in healthy adults. However, although pDCs in premature infants have TLR7 and TLR9 levels similar to adults, these cells secrete low concentrations of IFN-α/β, so that following viral exposure, IFN secretion is reduced and innate anti-viral immunity is not as effective as in adults (Schüller et al., 2013). A progressive increase in the numbers of cDC1s, cDC2s, and pDCs is observed during the transition from infancy to childhood, suggesting that a major immune remodeling process occurs early in life. Young adults and the elderly exhibit similar percentages of cDC subsets, and the dominant cDC subset in older adults is cDC2 (49%). The percentage of pDCs in adults was estimated at 28%. Hence, Nehar-Belaid et al. (2026) also reported that cDC1s expanded preferentially in adulthood, likely to compensate for the age-related decline in T-CD8^+^ cells. Finally, Reitsema et al. (2024) reported a significant effect of aging on DCs, so that a lower proportion of pDCs was observed in healthy older subjects versus healthy young individuals, which may explain the reduced capability of older people to fight viral infections.

### 2.4 Innate lymphoid cells

ILCs, an important first line of host defense, consist of a heterogeneous collection of tissue-resident lymphocytes that control tissue integrity, inflammation, immune regulation, and microbiome composition. Distinctive features are the absence of somatically rearranged T (TCR)s and B (BCR)s cell receptors and quick reactivity to local environment signals and cytokines. Based on their reliance on main transcription factors, secretory profile, and location, they have been subdivided into three main subtypes: groups 1 ILCs, 2 ILCs, and 3 ILCs. Group 1 ILC members are the ILC1 and NK cells which reside in the liver, rely on T-BET transcription factor, produce IFN-γ, and are critical for defense against intracellular pathogens and cancer cells. ILC2 representatives relying on GATA binding protein (GATA)3 expression are more abundant at mucosal sites in the skin, lungs, and gut, secrete IL-13 and IL-5, and ensure host protection against parasites. Finally, ILC3 members that express retinoid-related orphan receptor (ROR)γt secrete IL-17 and IL-22 and are responsible for defense against extracellular bacteria and fungi and the formation of secondary lymphoid tissues in embryos (Guendel and Lécuyer, 2025). From a functional perspective, ILCs may be broadly classified into cytotoxic-like ILCs that include NK cells and helper-like ILCs such as ILC1, ILC2, and ILC3 (Vivier et al., 2016).

NK cells are central to innate immunity due to their ability to exert important antiviral and antitumoral effector functions. In adults, the role of NK cells is to limit viral replication and dissemination before specific immunity is generated. At birth, newborn NK cells have reduced cytolytic function and are less sensitive to activation through receptors for IL-2 and IL-15, secreting lower levels of IFN-γ. Although NK cell activation is diminished, these cells still confer some neonatal antiviral protection (Lee and Lin, 2013). Regarding NK development and function during fetal and neonatal life, several aspects need to be emphasized. In early pregnancy, peripheral NK (pNK) and uterine NK cells are subjected to distinct phenotypic and functional modification in order to sustain fetal development. Briefly, the number of maternal pNK cells decreases as a consequence of their migration and differentiation into the decidua, giving birth to decidual NK cells, which are non-cytotoxic cells with enhanced secretory activity (IFN-γ, vascular endothelial growth factor (VEGF), and placental growth factor (PLGF)) that are critical for immune tolerance, angiogenesis, and normal fetal development (Arruvito et al., 2008). Due to their IFN-γ production, decidual NK cells are important immune regulators that act on inflammatory DCs and Th17 cells, thus promoting immune tolerance and the evolution of pregnancy. However, the hyperactivation of decidual NK cells should be tightly controlled, as it may result in spontaneous abortion and implantation failures (Adler et al., 2025; Guan et al., 2025).

NK cell maturation is characterized by the downregulation of CD56 and the acquisition of CD16 and NK cell immunoglobulin-like receptors (KIR), leading to the identification of three main subsets: immature (CD56brightCD16^−^), intermediate (CD56brightCD16dim), and mature (CD56dimCD16^+^) NK cells, each with distinct roles; among these, the mature NK subset exhibits the most elevated cytotoxic potential. Caba-Flores et al. (2024) reported that the NK cell phenotype in preterm milk is predominantly cytotoxic, thereby providing protection against infections for premature infants.

Immunophenotyping in newborns has revealed that CD56bright NK cells are the most elevated NK subsets and decrease during lifetime (Uyen Pham et al., 2022). However, with age, the phenotype of NK cells changes as the generation of mature NK cells is highly promoted (Manser and Uhrberg, 2016). Thus, aging leads to significant changes in the number of NK cells, subset distribution, phenotype, and effector functions. There are conflicting reports regarding NK cell count dynamics during lifetime (Hazeldine et al., 2012; Jin et al., 2021; Qin et al., 2016); however, aged NK cells are highly dysfunctional in terms of cytotoxic function, degranulation, and cytokine secretion, being less potent in eliminating senescent and malignant cells (Hazeldine et al., 2012). Thus, they favor chronic inflammation and increase susceptibility to associated pathological conditions such as cancer. Additionally, some reports highlight that dysfunctional NK cells may be linked with morbidity and mortality prediction in older individuals, independent of their age (Yang et al., 2026).

Although the classification of tissue-resident ILCs is well-defined and generally accepted, the situation is much more complex for the classification and roles of circulating ILCs (cILCs). These have been evaluated in both peripheral and umbilical cord blood, yielding valuable insights into their phenotypic and functional plasticity over the course of development. It appears that the frequency of cILCs in peripheral blood, mainly cILC1s, decreases during the transition from the neonatal stage to adulthood and old age; however, further studies are required to identify the factors responsible for these changes (Bennstein et al., 2020; Uyen Pham et al., 2022). Furthermore, major differences have been identified between cILCs in umbilical cord and peripheral blood—such as the reduced expression of transcription factors such as ID3 during the transition from neonatal to postnatal ILCs—suggesting that cILCs undergo major transcriptional and quantitative changes throughout life. However, recent progress in scRNA-seq technologies is expected to yield novel mechanistic insights into tissue-resident and cILC abundances and roles across the human lifespan (Bennstein et al., 2026).

### 2.5 Innate-like lymphocytes

Innate-like lymphocytes are another special, heterogeneous cellular population, present before or at birth, which aim to provide early life protection before the emergence of adaptive immune responses (Leila et al., 2025). NKT and γδ T cells are the most common unconventional lymphocytes; they bridge innate and adaptive immunity involving clonal expansion, although more limited than conventional T lymphocytes (Kaur, 2026; Ravens et al., 2017). This is in contrast with ILCs, which rely exclusively on germline encoded receptors and do not involve clonal selection and expansion for antigen recognition and activation (Eberl et al., 2015).

NKT cells are a subset of unconventional T cells that express both TCR and certain NK cell surface markers; these cells have limited TCR diversity and a narrow antigen recognition spectrum. NKT cells mainly reside in the liver and bone marrow. Upon activation, NKT cells can rapidly secrete a large amount of Th1 and Th2 cytokines, thereby exerting immunomodulatory effects. Additionally, NKT cells can promote cell-mediated immunity against tumors and infectious agents and can be involved in autoimmune diseases (Courtney et al., 2023). A recent study showed that the frequency of circulating NKT cells increases with age, independently of T-lymphocyte counts. Interestingly, when NKT cell subpopulations were quantified, an age-related increase in NKT-like/CD8^+^ cells was observed, alongside a decrease in NKT-like/CD4^+^ cells and double-positive cells (NKT-like/CD4^+^CD8^+^). For double-negative cells (NKT-like/CD4^−^CD8^−^), an upward trend was observed up to the age of 16, followed by a decline in adulthood. It could not be definitively determined whether these increases or decreases were associated with the percentages of T-CD4^+^ and T-CD8^+^ lymphocytes; however, it is suggested that the increase in circulating NKT cells in the elderly might compensate for the observed reduced frequency of T lymphocytes (Trombetta et al., 2025).

Another type of innate-like T cell is γδT cells. Unlike αβT cells, which are the important component of adaptive immunity, the TCR of these T cells consists of γ and δ chains. These cells have a phenotype predominantly CD4^−^CD8^−^ and constitute only 1%–10% of the total T-CD3^+^ cell population (Pistoia et al., 2018). γδT cells are distributed in mucosal and subcutaneous tissues such as the skin, small intestine, lungs, central nervous system, and reproductive organs, being part of the intraepithelial lymphocytes. γδT cells mainly recognize unprocessed peptide antigens and certain non-peptidic antigens presented by CD1 rather than antigen presented by major histocompatibility complex (MHC). Although representing a minor population, γδT cells are important components of nonspecific immune defense and play a particularly crucial role in local mucosal immunity and hepatic immune responses to infections, as well as in immune surveillance and homeostasis (Saura-Esteller et al., 2022). γδ T cells exhibit both innate and adaptive-like features, linking the two components of the immune system. While the study of γδ T cell ontogeny is an emerging field, recent studies have revealed a correlation between age and γδ T cell functionality in both experimental models and humans (Xu et al., 2020). Similar to other T lymphocytes, γδ T cells are generated in the thymus starting *in utero*; their production continues progressively through the postnatal period and childhood, but it declines with age. Interestingly, tissue-resident γδ T cells in children are characterized by high clonal diversity, which enables them to maintain homeostasis, ensure tissue protection, and acquire cytolytic properties with age (Jalali et al., 2022). Consistent with these observations, Nehar-Belaid et al. (2026) highlighted that peripheral γδ T and mucosal associated invariant T (MAIT, another unconventional lymphocytes) cell counts peak in childhood and young adulthood before declining significantly in older adults, thus validating a fluctuating pattern previously documented in clinical cohorts. This trajectory was particularly evident for peripheral Vδ2 γδ T cell subsets, which are known for their age-related remodeling.

Aging impacts innate immunity, and the main changes that are reported during this process will be further detailed and are presented in Figure 1. Aging significantly impacts Mo/Mf phenotype and functions, resulting in reduced phagocytosis and autophagy, while their number remains generally unchanged. Neu also exhibit lower phagocytic and chemotactic activities, with no changes in their count, while a lower proportion of pDCs was reported with aging. NK cells are also impacted by age. Thus, they undergo changes in phenotype, effector functions, and subset distribution and exhibit dysfunctional cytotoxicity, degranulation, and cytokine secretion. For innate-like lymphocytes, age-related changes have also been observed: for γδT cells, a downward trend was observed with age, whilst NKT cells increase until 16 years old, followed by a decline in adulthood.

**FIGURE 1:**
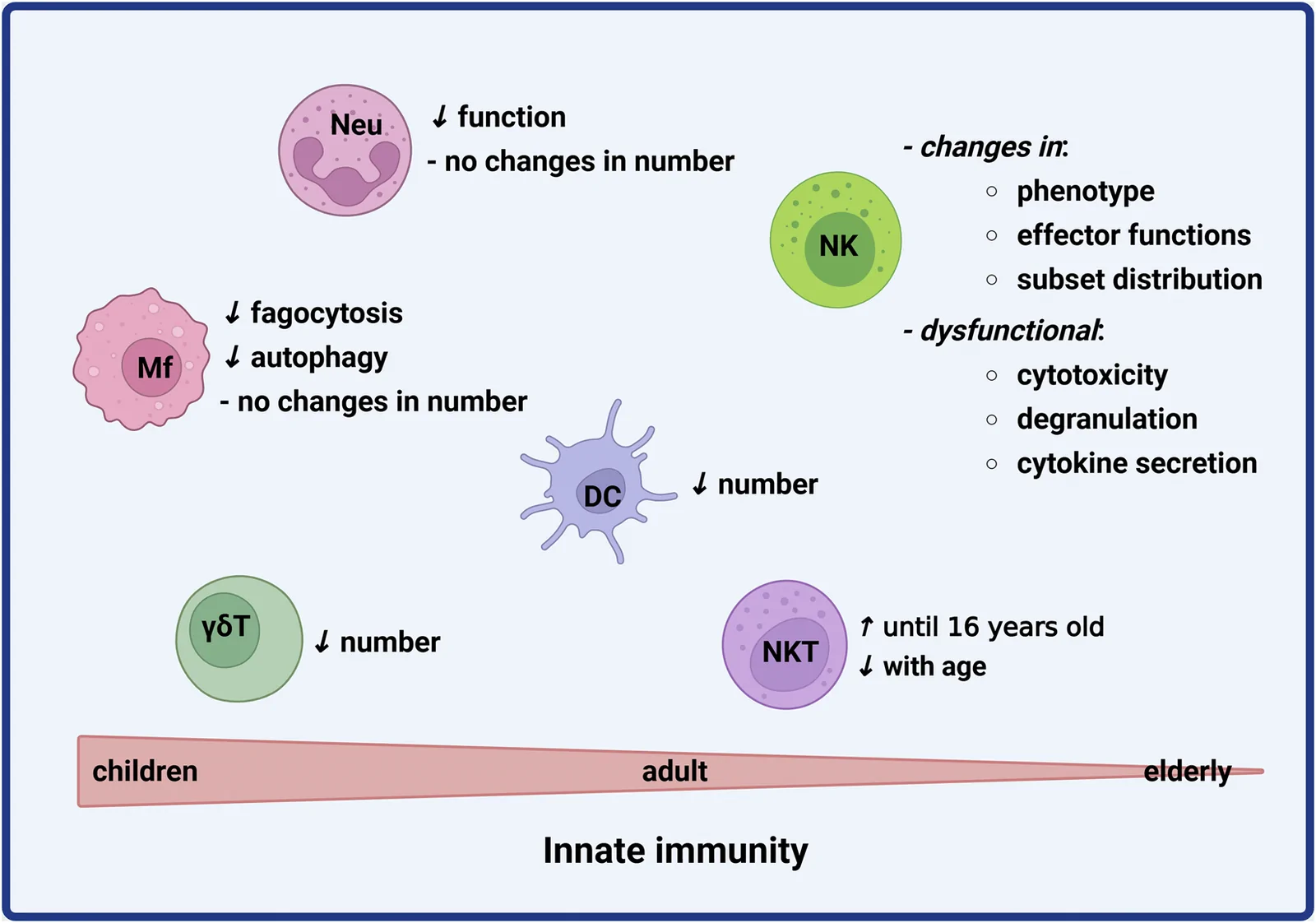
Innate immunity: The main changes undergone by innate immune cells throughout life. Created in https://BioRender.com.

### 2.6 Complement system

Another critical contributor to anti-microbial defense and inflammatory reactions is the complement system (CS). Most of the components that constitute the CS are synthesized by hepatocytes and are produced by tissue Mf, blood Mo, and epithelial cells of the genitourinary system and gastrointestinal tract. CS is activated through three independent pathways: i) the classical pathway (CP)—initiated when the C1q fraction binds to antigen-antibody complexes; ii) the alternative pathway (AP)—continuously activated by spontaneous hydrolysis of the C3 fraction, and also in the absence of Abs; iii) the lectin pathway (LP)—similar to the classical pathway, it does not require the presence of Abs and is activated by mannose residues from pathogen surface. CS fractions are involved in the opsonization of pathogens, are chemo-attractants for Mf and Neu, mediate microbial cell lysis that generates the complement membrane attack complexes (MACs), stimulate the release of inflammatory mediators, and influence the production of Abs. In newborns, the serum components of CS are 10%–80% lower than in adults and have low biological activity; their levels reach adult concentrations during the first month of life (e.g., B factor) (McGreal et al., 2012). In infants, the low levels of Igs make complement effector functions dependent on the AP and LP, which are initiated by polysaccharides and endotoxins—components of the bacterial wall. In adults, assessment of complement levels and activity in a gender-balanced, healthy Caucasian cohort revealed sex and age as critical determinant factors. Specifically, women exhibited lower CS activity and reduced levels of AP, LP, C3, and terminal pathway components than men. Additionally, CP and AP activity were significantly upregulated in older subjects, highlighting the importance of more elaborate studies when analyzing C-related pathologies and developing matched therapies (Gaya da Costa et al., 2018). Furthermore, C3 and C4 levels have been reported to increase with age and are associated with an elevated risk of metabolic syndrome and cardiovascular disease in older populations; in contrast, reduced C3 and C4 levels were observed in centenarians, correlating with healthy longevity and indicating a distinct proteomic profile that could be leveraged to prevent metabolic disturbances (Fu et al., 2020). In line with these observations, a recent study demonstrated that caloric restriction can modulate the C3a/C3 ratio, leading to its downregulation and the attenuation of age-related inflammation in humans, thereby highlighting C3a as a potential inflammatory checkpoint that can be targeted for the dietary modulation of inflammaging in clinical cohorts (Mishra et al., 2026).

## 3 Adaptive immune system

When the innate immune response is not sufficient to completely remove the pathogen, the adaptive (specific, acquired) arm of the immune system intervenes. There are clear differences between the two systems in terms of speed, specificity, and generation of long-term immunological memory (Figure 2; Table 1). Innate immunity is nonspecific, the response is short-lived and generally does not lead to immunological memory, while adaptive immune cells specifically recognize pathogens and confer long-term immunological memory.

**FIGURE 2:**
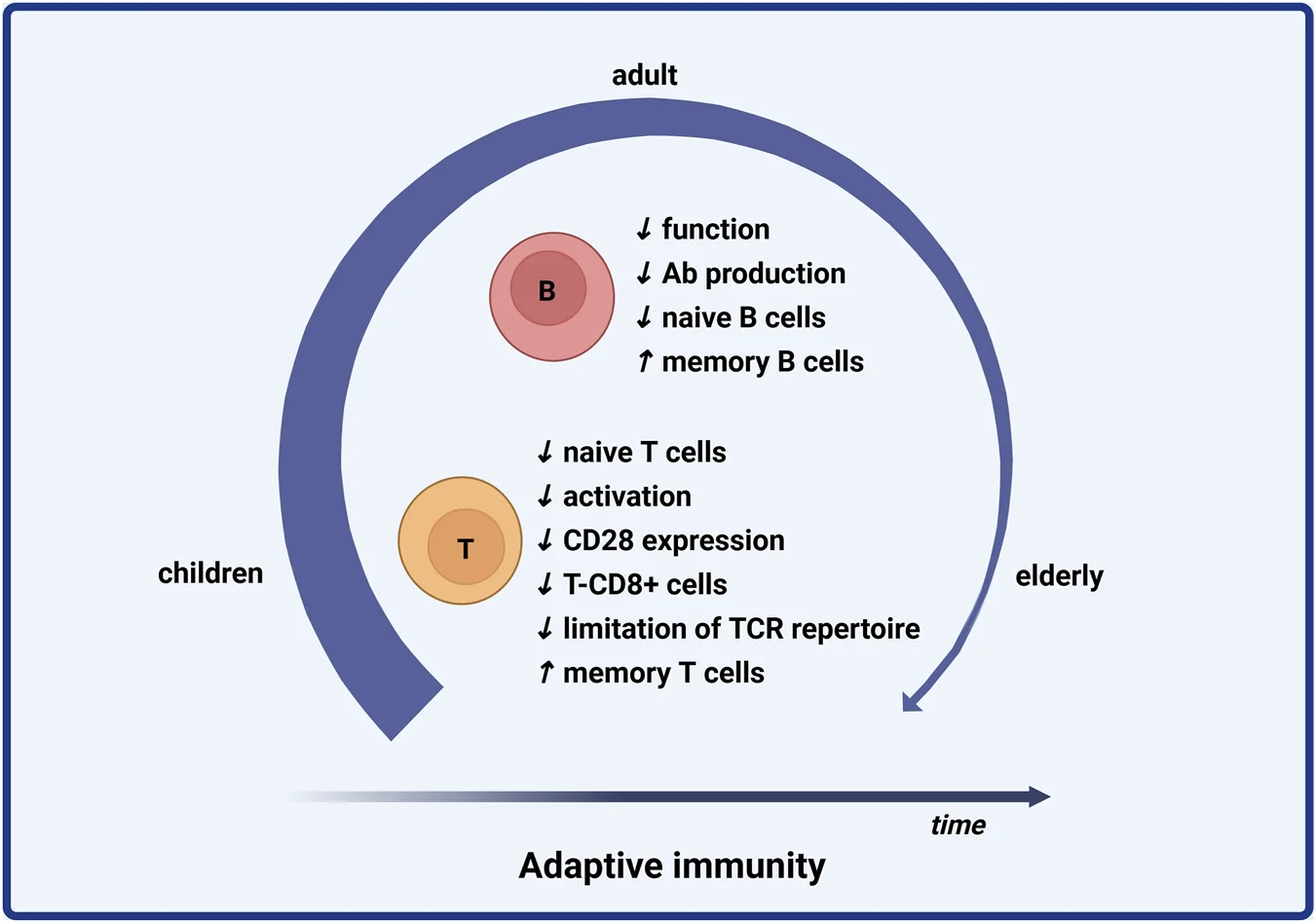
Cells involved in specific and nonspecific immune responses (adapted from Netea et al., 2019). Pathogen invasion determines the activation of innate immunity (Mf, NK cells, humoral factors, etc.), followed by the specific immune response, the final result being the elimination of the pathogen and immunological memory induction. Created in https://BioRender.com.

**TABLE 1:** Differences between innate and adaptive immune systems.

Innate system	Adaptive system
In phylogeny—first present in invertebrate metazoans	In phylogeny—first present in jawed vertebrates
Active since birth	Active after 3 months since birth
Includes physical barriers—skin, mucous membranes, associated secretions (saliva, gastric juice, tears, lipid skin secretions, etc.)	—
Quick response—minutes–hours	Delayed response—days–weeks
Non-specific antigen recognition	Specific antigen recognition
Multiple mechanisms of action, multiple cell populations to cover the first line of defense	Reduced mechanisms, well controlled/regulated, limited and specific cell populations
Limited immunological memory	Specific and sometimes lifelong immunological memory

Although the specific and innate mechanisms initially seemed to function separated in time, there is an efficient network between the two systems through which cells and humoral components are interconnected and influence each other. There are four mechanisms by which innate immunity triggers an adaptive response. The first signal is initiated either by antigenic peptides presented by MHC and recognized by the TCR or by Ag binding to BCR without MHC involvement. The antigenic peptides are exposed by cells of the innate system. The second mechanism is composed of immune checkpoint (IC) molecular pairs. Cytokines are the third type of signaling. The last type is more recently discovered, and its main idea is that metabolism-associated danger signals (MADSs) are recognized by metabolic sensors (Dai et al., 2017). Although some studies have found that adaptive immunity can be independent of innate immunity, such as type II immune responses triggered by allergens and parasites, innate immunity still plays a major role in activating adaptive immunity.

The activation of the adaptive immune response and induction of immunological memory depend on the nonspecific immune system, the downstream effects being the amplification of phagocytosis and destruction of the pathogen by specific innate immune cells.

The two forms of immunity are differentiated by their specificity and ability to induce long-term immunological memory. The innate immune response is non-specific and without adaptation mechanisms, while the specific immune response recognizes with great precision a multitude of pathogens using the recombination mechanisms of Igs family genes (Danilova, 2012), thus achieving long-term immunological memory.

The development and maturation of the adaptive immune system during early life is crucial for establishing immune tolerance and responding to pathogens. The developing immune system must learn to differentiate between commensal microbes and pathogens—“immune education”.

Early life interactions with the gut microbiome significantly influence this process, as the inflammatory milieu of the microenvironment mediates information flow from pre-weaning periods to adulthood (Tepekule et al., 2025).

Research indicates that fetal immune cells develop as early as the fourth week of gestation, and mature T and B lymphocytes can be detected by the end of the first trimester. Understanding these dynamics is essential for developing interventions that protect against infectious diseases and inflammatory disorders in early life. These insights highlight the importance of early life experiences in shaping the immune system’s functionality and resilience.

Upon the development of an adaptive response, specific cells endowed with immunological memory confer a long-term host protection against the same type of pathogen, and upon subsequent contact the immune response is much more extensive, developing faster and with higher accuracy.

The fundamental characteristic of the adaptive immune system is the recognition and destruction of a non-self that can be an actual pathogen, its secreted molecules, or a self-cell/tissue that has gained non-self-characteristics. It is essential that the adaptive responses be directed to foreign molecules and not to self molecules. When the immune system fails to make the correct identification, autoimmune diseases can occur—some having lethal potential.

The acquired immune response is highly specific and is conditioned by the body’s previous contact with the pathogen through a natural infectious process, active immunization (vaccination), or through the transfer of Abs from an immune donor (passive immunization) (Neagu et al., 2021a). However, specific immunity is relative because it can be defeated by the aggressiveness and virulence of a large number of infectious agents and/or by their antigenic versatility.

The adaptive immune system performs three major functions. a) Specific recognition of non-self Ag during the Ag presentation process: Ag-presenting cells (APCs) (e.g., DCs) are activated and present antigenic peptides to naive T lymphocytes from lymph nodes in MHC restriction. The presence of MHC assures the immune system that the presented peptides are “controlled” by the self-machinery. b) Elimination of the pathogen by itself or the infected cells with the pathogen: T lymphocytes migrate to the infected inflammatory site, where they release inflammatory mediators in coordination with all the immune cells that were already attracted by the inflammatory site, thus activating and maintaining the immune response until the total elimination of the pathogen. c) Development of immunological memory: during the first encounter with a specific Ag, the adaptive immune system also generates a small population of cells that sustain the future immunological memory if needed for a second encounter with that Ag. The existence of a small pool of memory cells is proven by the fact that Abs generated following the attack of an infectious agent can still be detected in the circulation in small quantities, such as anti-MERS Abs detected even years from infection (Li et al., 2020).

### 3.1 T lymphocytes

Derived from hematopoietic progenitors in the bone marrow, T lymphocytes complete their development and maturation in the thymus (7–8 weeks of gestation), from which they are subsequently exported into the periphery (12–13 weeks of gestation). There are three main peripheral T-lymphocytes subsets: 1) naïve T cells, quiescent cells that are able to recognize and respond to new Ags; 2) memory T cells, or antigen-experienced T cells, that shape long-term immune surveillance; 3) regulatory T cells (Tregs) that regulate self-tolerance and homeostasis (Mena et al., 2026). Simultaneous with the involution of the thymus, there is a progressive decrease in naïve T lymphocytes in humans, which is more significant over 60 years (Palmer et al., 2018). Conversely, the proportion of antigen-educated T cells increases with age: while newborns have only 1% of educated T cells, in 60+ adults they have 65% of total T cell fraction (Nian et al., 2019). In addition, with advancing age, a pronounced limitation of the TCR repertoire was observed, which cannot be counterbalanced by the proliferation of naïve T lymphocytes, leading to an altered immune response to new antigens (Nian et al., 2019). T cell activation, proliferation, and survival are closely linked to the expression of CD28, a key costimulatory molecule expressed on all T lymphocytes at birth. CD28 loss has been reported on both T-CD4 and T-CD8 cells with aging, with more than 50% in T-CD8^+^ lymphocytes (Weng et al., 2009). In parallel, Goyani et al. (2024) reported the reduction of T-CD8^+^ cells and T-CD45RA^+^ naïve cell frequencies, while the proportion of T-CD4^+^ was not affected by aging. However, it has been noted in older individuals that naive T-CD8^+^ and T-CD4^+^ cells correlate with immunosuppression as they are less potent in response to novel Ags. Nehar-Belaid et al. (2026) emphasized the conversion of naïve to memory phenotypes with age, this trend being more pronounced for T-CD8^+^ cells. The same study reported a high prevalence of SOX4^+^ naïve T-CD4^+^, and T-CD8^+^ cells in infants (∼20% of total naïve T cells), which diminished rapidly with aging. The impact of aging on the abundance of Tregs remains controversial, with some studies indicating a decline of the proportion of these cells resulting in a weakened immune system (Doherty et al., 2025; Neagu et al., 2025). However, most studies show that the percentage of Tregs may increase or remain stable with age, although their function is altered (Lages et al., 2008; Palatella et al., 2022; Rosenkranz et al., 2007).

The dysregulation of T cells has been reported with aging, which has prompted further research on a potential correlation between T cell immune responses and increased susceptibility to infections in the elderly. These were conducted mainly due to the role that memory T-CD4^+^ cells play in B cell activation, class switching, and affinity maturation. Gong et al. (2025) studied 300 younger and older adult subjects (aged 20–90) and reported significant, non-linear transcriptional reprogramming in T cells with aging, independent of chronic cytomegalovirus infection or systemic inflammation. Thus, a Th2 polarization of T cells was reported during aging that was associated with dysregulated B cell responses, altered antibody class-switching, and a reduced neutralizing capacity of antibodies to antigens contained in influenza vaccines. These observations may explain the lack of response to vaccination and increased susceptibility to influenza infections in the elderly, which can lead to death in 70%–85% of cases (Pires, 2025).

### 3.2 B lymphocytes

B cells are major contributors to adaptive immune responses because they produce antibodies and cytokines and act as APCs to enhance T cell activation. B cells represent a highly heterogeneous cellular population that develops *in utero* and undergoes a succession of transformations following exposure to the extrauterine environment. B-cell precursors have been reported in the fetal liver as early as the seventh week of pregnancy; subsequently, from the second trimester through the postnatal period, B-cell development occurs in the bone marrow, which becomes the primary site of B lymphopoiesis and where immunoglobulin (Ig) or B-cell receptor rearrangement takes place. Immature B cells expressing IgM at their surface exit the bone marrow and enter the circulation, from where they migrate to secondary lymphoid organs such as the spleen and lymph nodes. Here, they undergo complex processes, including class switching, somatic hypermutation, and affinity maturation, that are essential for B-cell maturation and the generation of memory B cells and long-lived, antibody-producing plasma cells (Borriello et al., 2022). In contrast to plasma cells, which migrate to bone marrow, memory B cells (generated following an Ag encounter) remain in the periphery, where they constantly patrol between the blood and lymphoid organs, contributing to the formation of long-term immunological memory (Piatosa et al., 2010).

Interestingly, a meta-analysis of 28 studies focusing on the assessment of B-cell levels in healthy infants and the determination of reference values during the first year of life revealed a dynamic pattern: an initial drop in B-cell levels from cord blood during the first week of life, followed by rapid expansion over the next 2 months, peaking at 6 months, and then a subsequent gradual decline up to 12 months, reflecting profound immune system remodeling in response to external Ags exposure (Borriello et al., 2022). Particular attention should also be paid to the general characteristics of umbilical cord blood B cells. Although these cells have traditionally been described as immature, relatively recent studies highlight that they actually possess a mature phenotype similar to that of adult B cells. Thus, it seems that umbilical cord blood B cells are neither dysfunctional nor immature; rather, they differ from their mature adult counterparts in their highly conserved BCR repertoire, efficient IgA class switching, and rapid immune responses, thereby compensating for the low numbers of memory B cells and reduced Ab titers observed in neonates (Budeus et al., 2021).

In newborns, naive (CD19^+^IgD^+^CD27^−^) and transitional B (CD19^+^IgMhighCD38high) lymphocytes that are not Ag-experienced cells predominate (Piątosa et al., 2010). However, age-dependent maturation was observed in circulating B cells in children as portrayed by increasing rates of somatic hypermutation in their BCRs (Nielsen and Boyd, 2019). With aging, a decrease in naïve B cells has been observed, concomitant with an increase in memory B (CD19^+^CD27^+^) lymphocytes; however, Sopena-Rios et al. (2026) studied over 980 healthy subjects and highlighted that men display elevated levels of naïve and intermediate B lymphocytes compared to women, providing a rationale for incorporating sex into strategies aiming to prevent immunosenescence.

B cells are specialized immune cells that excel at modulating humoral immunity. In human newborns, all circulating IgGs antibodies are of maternal origin and transferred across the placenta to provide passive immunity until newborn IgG production takes over 15 weeks after birth. However, maternal IgG can also negatively interfere with newborn vaccine responses. The concentration of IgG increases sharply during the third trimester of gestation, and preterm children are believed to largely lack this passive immunity (Pou et al., 2019). Maternal immune cells transferred to the developing fetus during pregnancy impact the developing immune response of the fetus and neonates and even persist for decades after birth.

Breast milk extends the maternal immune influence into the postnatal period. The most well-studied immunological components in breast milk are Abs. The concentration and proportion of Abs change over the course of lactation from colostrum in the first 48 h of lactation to more mature milk by 10–15 days postpartum (Langel et al., 2022).

Memory clones represent the actual effectors of a secondary immune response. Thus, a small number of T and B lymphocytes become memory cells and will immediately recognize Ags in a secondary immune response, similar to the primary response. The primary response is characterized by low amplitude in terms of the Abs concentration, duration of the specific Abs secretion, and intensity of the Abs neutralizing action. Subsequent exposure to the same Ag induces the secondary immune response, which is more prompt, intense, refined (Abs have a higher affinity and specificity toward the Ag) and sustained over a longer time than the primary immune response. Thus, due to the genetic rearrangements within memory B clones, the secondary response Abs have an increased affinity towards the pathogen Ag.

Abs can directly bind to the free viral particles, thus blocking the subsequent infection of host cells. With this action, B cells actually mark the pathogen that is enclosed in an array of specific Abs to be cleared by cells having FcR that will engulf the pathogen. T lymphocytes act differently than B cells, recognizing specific non-self Ags after antigenic peptide presentation. A subpopulation of T-CD4^+^ cells will be activated, coordinating the action of several other cells through the array of cytokines/chemokines generated. Another important subpopulation, T-CD8^+^ cells, destroy the infected or abnormal cells that display specific markers. Analogous to B cells, T cells also develop memory lymphocytes that safely and more efficiently guard upon re-infection with the same pathogen.

Aging impacts adaptive immunity, and the main changes described above are summarized in Figure 3. Thus, aging leads to a decrease in naive T and B lymphocytes, while both memory T and B cells increase with age. A decrease in antibody production has also been reported, and in the case of T cells, a pronounced limitation of TCR repertoire was observed. Aging also causes a diminished CD28 expression on both T-CD4 and T-CD8 cells, as well as a decrease in the number of T-CD8 cells. The adaptive immune response differs from the innate host defense by specificity, immunological memory, and the ability to distinguish between self and non-self. Specificity is the property of Abs and T lymphocytes to discriminate between related Ags, while immunological memory represents the ability of the adaptive immune system to respond in an improved manner upon second exposure to an identical Ag.

**FIGURE 3:**
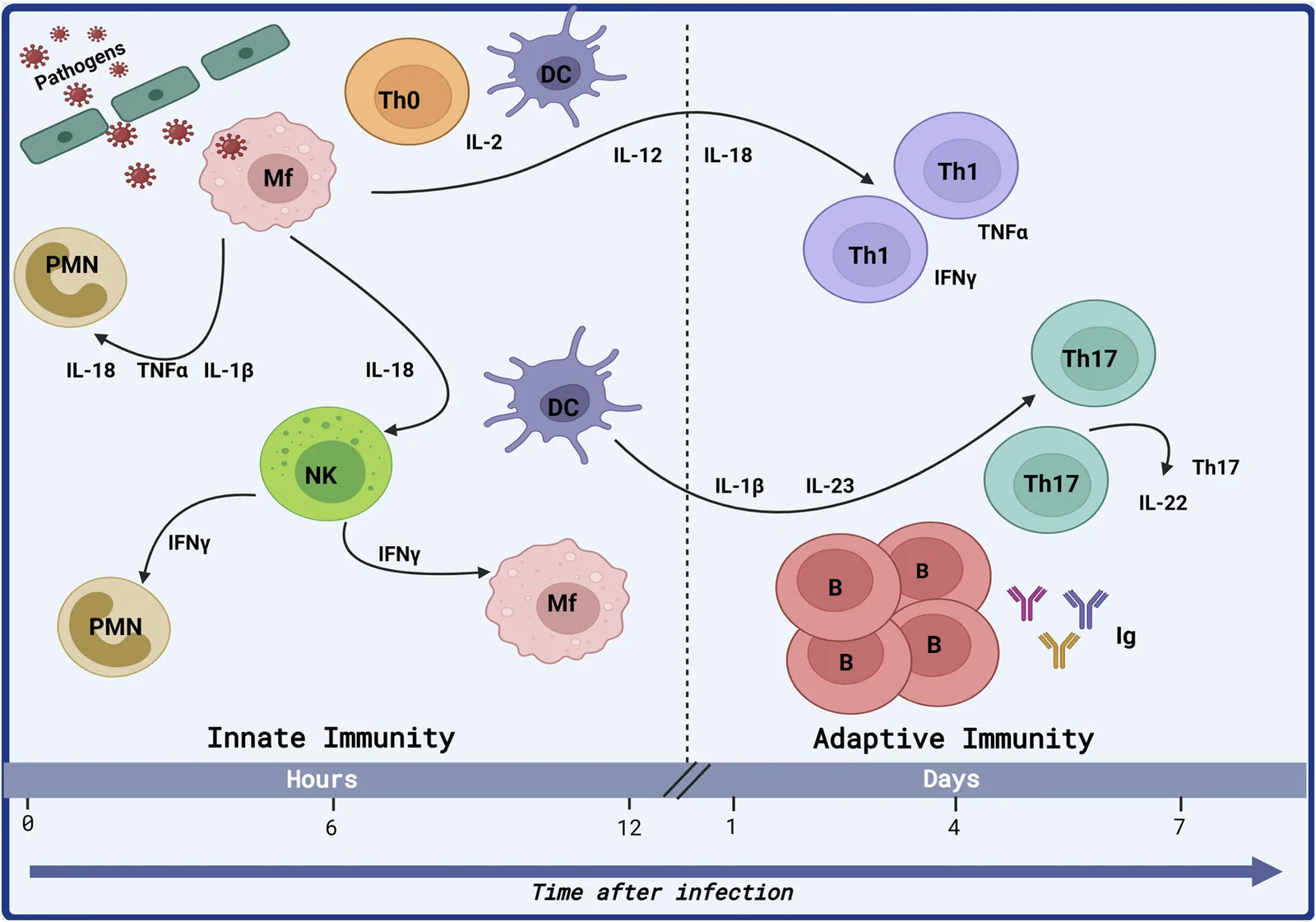
Adaptive immunity. The main changes undergone by adaptive immune cells throughout life. Created in https://BioRender.com.

## 4 Immunological memory

Immunological memory is one of the finest examples of the evolution of the immune response. After the inflammatory immune response to danger-associated Ag, some of the Ag-specific T and B cells persist in the body and become long-living memory T and B cells. After the second encounter with the same Ag, they recognize the Ag and mount a faster, more robust response. Immunological memory is also the basis of vaccination.

Emerging resources show that even the innate immune system can initiate a more efficient immune response and pathogen elimination after the previous stimulation with a pathogen, respectively, with pathogen-associated molecular patterns (PAMP)s or damage-associated molecular patterns (DAMP)s. Innate immune memory, or “trained immunity”, is neither Ag-specific nor dependent on gene rearrangement; the different response is caused by changes in epigenetic programming and shifts in cellular metabolism (Gourbal et al., 2018).

Newborns have immunological help from two sources: placental transfer before birth and milk after birth. The passive protection that the maternal trans-placental mechanisms furnish is short-lived and decays by the time a child is about 6 months old. For example, the estimated duration of protection by maternal Abs among infants is 3.3 months for measles, 2.7 months for mumps, 3.9 months for rubella, and 3.4 months for varicella. In neonates, the cellular immune system matures rapidly in the first 3 months of life, with multiple factors that influence this maturation process. The mother’s nutritional imbalance, both deficiency and excess, can have a considerable effect on neonatal immunity at birth and immune maturation in early life. Human milk contains immunomodulatory cells and cytokines that protect newborns and infants from respiratory infections such as respiratory syncytial virus, bronchiolitis, and allergies. Human milk also contains lactoferrin, an iron-binding glycoprotein that is important for innate immune host defenses at birth because it exhibits broad-spectrum antimicrobial activity and prevents invasive fungal infections (Manzoni et al., 2012).

There is clear adaptive response immaturity in neonates, which is why they exhibit increased susceptibility to infections. The immaturity in their lymphocytes, including a high frequency of naïve recent thymic emigrants, low numbers of effector-memory T-cells, impaired Th1 cytokine secretion, and reduced strength of BCR signaling, sustain this overall adaptive immune immaturity. However, neonates are capable of mounting adult-like T and B cell responses, but with important qualitative and quantitative differences. Furthermore, neonates have underdeveloped germinal centers in their lymph nodes and spleen and low expression of B-cell receptors, including CD21, CD40, CD80, and CD86, all resulting in low levels of primary IgG responses to infections and vaccines (Basha et al., 2014).

Since the 1990s, the main focus of research in the immunological memory domain has been the immune response to viral infections. These studies have frantically continued since the emergence of the SARS-CoV-2 pandemic (Neagu et al., 2021b).

Although immunological memory was long considered to belong exclusively to the adaptive immune system, recent research has shown that the nonspecific immune system can also respond to previously encountered Ags. This has been demonstrated in invertebrates without adaptive immunity by the existence of protection against reinfection—a property called “trained immunity” (Morita, 2021). This provides an increased nonspecific immune response for several months, without the production of specific Abs; it is a primitive adaptative system, mediated by epigenetic changes and alterations in cellular function without modifying the DNA structure. Nonetheless, trained immunity has been also associated with chronic inflammatory disorders such as autoimmune diseases, allergies, and atherosclerosis (Kim and Lee, 2025).

Mo, resident Mf, NK, and bone marrow precursor cells can be subjected to trained immunity. Although the memory is not as specific as is the case for T or B lymphocytes, specific members of pattern recognition receptor (PRR) families induce intracellular signaling cascades that contribute to innate immune memory. Among those PRRs are dectin-1, the receptor for β-glucan, and NOD2, the receptor for muramyl peptide, a molecule from the cell envelope of the bacterium *Mycobacterium bovis*. It has also been proposed that nonimmune cells, such as fibroblasts, that play a supportive role in immunity can also be trained. This immunological memory imprinted in cells of the innate immune system occurs through metabolo-epigenetic modifications (Saeed et al., 2014).

A key role in immunological memory is played by B lymphocytes. In viral infections, long-lived plasma cells are generated which secrete various Igs that are specific to the encountered Ags. These memory-driven Igs have higher affinity for the re-encountered Ags, and even their specificity is more refined than those generated in the primary response. This immune memory is largely mediated by the two main components of B cell memory (MBCs) and long-lived plasma cells (PCs). The chemical and cellular mechanisms that encourage fat selection for MBCs or long-lived PCs are an area of active research. A deeper understanding of the specific cellular and molecular pathways that govern the generation, function, and reactivation of MBCs is critical for overcoming the challenges associated with vaccine development (Syeda et al., 2024).

In addition to B lymphocytes, memory T-helper lymphocytes are also involved in immunological memory; these latter represent a heterogeneous cellular subpopulation in terms of location and function. In peripheral lymphoid organs, naive T-helper lymphocytes are activated by antigenic peptides presented by APCs followed by their differentiation into functional effector T-helpers (Th1, Th2, Th9, and Th17), each subset secreting cytokines involved in the processes of eliminating various types of pathogens (Hirahara et al., 2021).

These subsets of Th are differentiated by their secretory pattern. Th1 lymphocytes produce large amounts of IFN-γ to eliminate intracellular bacterial pathogens (e.g., *M. tuberculosis* and *L. major*). Th2 subset produces IL-4, IL-5, and IL-13, which are essential in the fight against parasites, and the Th9 subset secretes IL-9 in anti-helminthic activity. Th17 group secretes IL-17A and IL-17F, with a defense role against extracellular fungi and bacteria (e.g., *K. pneumonia*). These effectors coordinate the Neu and eosinophils to inflammatory sites and secrete proinflammatory cytokine necessary to initiate an inflammatory response that destroys the pathogen.

Similar to T and B lymphocytes, NK cells can exert immunological memory after encountering different stimuli (haptens or viruses), resulting in the generation of Ag-specific memory NK cells. NK cells can also “remember” their inflammatory cytokine environment that imprints long-lasting, non-Ag-specific effector function. Although NK cells are generally prepared for rapid cytolytic activity, in many cases they must be functionally differentiated or primed by cytokines or other immune cells, such as DCs, to achieve optimal effector responses. NK cell activation is determined by a delicate balance established between signals provided by activating receptors, which recognize stress-induced ligands on virus-infected cells, and inhibitory receptors, which predominantly engage MHC class I molecules. Effector and memory T lymphocytes could support NK cell memory responses by providing IL-2, which further stimulates NK cell expansion and supports memory. Recent discoveries regarding the generation of memory NK cells in viral infection could have a major impact on novel antiviral vaccination strategies (T-cell-based strategies). The mechanisms by which memory NK cells contribute to anti-viral protection are not fully understood. Memory NK cells, with enhanced effector functions, may cooperate with effector T lymphocytes and Abs to provide faster and more efficient control of viral infection. It is also possible that memory NK cells may support memory T lymphocyte responses by rapidly providing inflammatory cytokines and chemokines that further orchestrate the migration and function of adaptive immune cells (Sheppard and Sun, 2021).

NK cells in newborns are among the earliest cellular responders to viral infection. Because their signaling paradigm is combinatorial, acting through a spectrum of activating and inhibitory receptors, NK cell phenotype and function are uniquely and closely linked. Interesting results have been reported by comparing adult circulatory NK phenotype with NK cells from umbilical cord blood. Cord blood NK cells have a less mature phenotype than adult NK cells, but the proportional abundance of the CD56bright and CD56dim subsets does not differ between these groups (Strauss-Albee et al., 2017).

Several features of immunological memory are also found in Mf. Early studies showed that Mf could be plastic and “adaptive”: they can modify the expression of recognition receptors for a wide range of pathogens, enhance effector functions, and alter the regulation of the inflammatory response. In addition, it has been shown that Mf could be trained by lipopolysaccharides to become more or less responsive to subsequent activation signals (Kloc et al., 2023).

Recent studies have shown that Mo and Mf exposed to *C. albicans* or β-glucans exhibit an enhanced response (mediated by epigenetic reprogramming) to subsequent stimulation with unrelated pathogens or pathogen-associated molecular patterns (Saeed et al., 2014).

The immaturity of neonatal Mf becomes evident with ongoing phenotypic differentiation and still emerging efficiency in movement, phagocytosis, and regulation of inflammation. These cells can be stimulated by an array of endogenous triggers, such as cytokines, oxidized lipids, reactive oxygen (ROS) and nitrogen (RNS) species, metabolic products, and debris released from dying cells, such as heat-shock proteins and DAMPs (de Jong et al., 2023). In infants, Mf production and activation is well regulated and maintained at low physiological levels. However, during stress, infection, and inflammation, the Mf population may be expanded, having an altered proportion of various subsets. The Mf subsets may also be altered when inflammation begins to resolve and during the restoration of tissue architecture or scarring. In other instances, immature Mf recruited during resolving inflammation may trigger apoptosis, persistent low-grade inflammation, or atypical inflammatory and microvascular changes (Yang et al., 2020).

γδ T lymphocytes have the ability to acquire memory characteristics, including accelerated activation and proliferation upon re-encounter with a stimulus. Importantly, each type of “trained” γδ T cell appears to retain core properties of its precursor with respect to homing and cytokine secretion. γδ T cells play a crucial role in the immune system of newborns, particularly in the context of sepsis. They are a unique subset of T cells that develop early in fetal life and can respond to non-peptide Ags, providing rapid protection against pathogens. In pre-term infants, γδ T cells are particularly important due to their high activity and ability to produce cytokines that help combat infection. During the neonatal period, they are essential for establishing immunity as the infant transitions from the protective maternal environment to the outside world. Research indicates that γδ T cells exhibit significant functional diversity and can be activated in response to infections, making them vital for the newborn’s immune defense. Overall, γδ T cells are integral to the newborn’s immune response, especially in the context of infections and sepsis (León-Lara et al., 2024).

There is increasing evidence that the development and function of ILC3 can be modulated by the maternal and neonatal microbiome and is involved in neonatal disease pathogenesis. ILC3 has a critical role in resistance to infection and disease pathogenesis in the newborn, with a focus on microbial factors that modulate ILC3 function involved in immunological memory (Mirpuri, 2021). ILCs can recall previous activation, respond more effectively during secondary encounters with allergens, and provide an enhanced secondary response similar to T lymphocytes, the main difference being the method of activation. T lymphocytes are activated by binding their TCRa to specific Ag presented by MHC molecules on APC cells, resulting in clonal expansion and the generation of Ag-specific memory T lymphocytes. In contrast, ILCs are activated by cytokines, resulting in Ag-nonspecific memory ILCs. However, ILC2s show an enhanced response similar to T lymphocytes (Favaro et al., 2022).

Recent data show that immunological memory is not restricted just to adaptive immunity mediated by B or T lymphocytes, as features of immunological memory have also been demonstrated in innate immune cells (Netea et al., 2016).

Significant recent progress has been made in understanding trained immunity in different age groups. The basic prototype of trained immunity studies remains the administration of live attenuated vaccines such as the *Bacillus* Calmette-Guérin (BCG) vaccine which confer long-lasting protection against subsequent infections with unrelated pathogens as a consequence of the functional reprogramming of myeloid cells. Trained immunity can also be induced in human and adult murine cells by the administration of PRR agonists such as β-glucan or LPS. Interestingly, in a recent scRNA-seq study in experimental murine models, Rincon et al. (2025) revealed that BCG vaccination at birth improves survival in murine neonatal sepsis and is accompanied by a profound remodeling of monocyte and neutrophil subsets, enhanced antimicrobial control, attenuation of proinflammatory signaling, and stimulation of T cell proliferation. Thus, administering the BCG vaccine shortly after birth entails a profound remodeling of the myeloid compartment to ensure effective, prompt responses to a future multi-agent infection. Fernandez et al. (2026) found that, in contrast to previous observations in adult mice, some PRR agonists may fail to induce trained immunity against *Leptospira* in neonatal mice, underlining the need to reflect on the risks and adverse effects associated with the administration of immunostimulants in the early stages of life. In adults, scRNA-seq analysis of BCG-induced trained immunity in 156 subjects revealed heterogeneous transcriptional states in monocytes and T cells in response to LPS stimulation. However, IFN-γ signaling and STAT1 were identified as key hallmarks of the trained monocyte subsets in BGC responders, whereas the disruption of these programs was linked to sepsis; thus, the molecular characteristics of type I IFN-related monocytes may be harnessed in the future for the development of innovative, immune-based therapies in patients with sepsis (Li et al., 2023).

The concept of trained immunity holds even greater significance for the elderly; in addition to debilitating neurological or cardiovascular conditions, these individuals exhibit increased susceptibility to infections. Vaccines remain the cornerstone of infection prevention in the elderly due to their accessibility and safety profiles; however, their efficacy is often questionable in this age group given the immune system’s reduced capacity to respond to immune stimulation. This is attributed to a decline in the pool of naive adaptive immune cells, as well as a diminished capacity for phagocytosis, chemotaxis, and cytokine production among innate immune cells (Bulut et al., 2020). Interestingly, it has been demonstrated that 2 months after BCG vaccination, individuals in Guinea-Bissau aged 50 or older produced higher levels of IFN-γ in response to *M. tuberculosis* stimulation and increased quantities of IL-1β, IL-6, and TNF-α in response to non-specific stimuli compared to control, suggesting that it may be possible to induce trained immunity in the elderly to provide protection against infectious diseases (Berendsen et al., 2024). In addition to these immunomodulatory effects of BCG in the elderly, there are also reports indicating that the BCG vaccine can mitigate the adverse effects of inflammaging by reducing levels of circulating pro-inflammatory markers (Bulut et al., 2020). However, despite these promising results, several recent studies on larger clinical cohorts report no stimulatory effect of BCG vaccination on the response to influenza vaccination (Madsen et al., 2026); furthermore, no protective effect of BCG vaccination against non-mycobacterial infectious diseases or other immune-mediated diseases was observed in a Dutch clinical cohort during the 2 years following vaccination. However, this last study emphasized that BCG vaccination could aggravate existing cardiac arrhythmias, raising concerns regarding its use in patients with arrhythmia (Dulfer et al., 2024).

## 5 Ageing of the human immune system

Maintaining homeostasis is another important task of the immune system. Besides eliminating aggressors, the immune system is also involved in post-aggression tissue repair, always maintaining the equilibrium between self and non-self. However, advanced age associates with chronic inflammation and immunosenescence, which orchestrates a multi-parameter remodeling of immune system that increases vulnerability to infections and to various age-associated disorders such as cancer, cardiovascular diseases, autoimmune diseases (e.g., diabetes), or neurodegeneration (e.g., Alzheimer’s disease, dementia).

Different mechanisms are involved in the immunosenescence complex process. Immunosenescence is driven by alterations in both the number and relative balance of T- and B-lymphocyte populations. These changes consist in the reduction of naive adaptive immune cells, the increase of memory T cells, and reduced ability to respond to new aggressors. No reduced T cell proliferation or capacity of APC are associated. Furthermore, age-related changes also appear in the innate immune system through reduced chemotaxis, reduced cytotoxicity, and abnormal cytokine production.

In the elderly, the consequence of reduced thymic production is a reduced number of naive T lymphocytes; although they proliferate and increase the “virtual memory” compartment, their ability to achieve immunological memory in response to *de novo* Ags is reduced. Cytokine production of T-CD4^+^ and T-CD8^+^ lymphocytes is altered, as is the expression of key surface markers, and the ratio T-CD4^+^/T-CD8^+^ cells is inverted. Regarding T-CD4^+^ lymphocytes, age-related changes include decreased proliferative capacity, inadequate differentiation of the Th subpopulation, and increased Treg percentage (Lorenzo et al., 2018).

Age has also been documented as having a significant impact on B cell subset prevalence and their functions. Briefly, naive B lymphocytes are replaced by memory cells, which remain quiescent in the circulation, even for decades, while waiting to encounter again the Ag that generated these clones. The expansion of age-associated double-negative (IgD^−^CD27^−^) B cells, which are linked to inflammation and diminished self-tolerance along with reduced overall B cell and switched B cell counts reductions, has also been noted in aged individuals. Additionally, aging has been documented to impair germinal center reactions and the proper functionality of T follicular helper cells, leading to impaired BCR signaling, reduced class-switch recombination, and somatic hypermutation (SMH) (Anaya et al., 2026). Thus, in the elderly, memory B cells have decreased affinity and isotype switching, leading to a decreased response to new Ags. This last assertion characterizes the overall phenomenon of immunosenescence. In general, changes in T and B lymphocytes prevent the development of an adequate immune response to new viral acute and latent infections but also to vaccinations (Garnica et al., 2022).

The alteration of the immune system with age can compromise the homeostatic balance between microbiota and the host. As an integral part of the dynamic organism throughout our lifetime, the gut microbiota co-evolves with age, drives the maturation of our immune system, and thus contributes to host health (Gao et al., 2024). Changes in the gut microbiota with ageing may elevate the expression of pro-inflammatory factors, contributing to chronic age-related inflammation. For example, in the absence of adequate short-chain fatty acid production, the permeability of the intestinal mucosa increases, allowing intestinal bacteria to pass through and enter the bloodstream. PAMPs of invading bacteria (e.g., LPS) bind to pattern recognition receptors expressed by immune cells and adipocytes and trigger the production of proinflammatory cytokines, leading to chronic inflammation (Di Vincenzo et al., 2024).

Given the role of the immune response in the control of tumor diseases, reduced or less efficient immune functions in the elderly contribute to increased morbidity/mortality. With ageing, a deregulated hematopoiesis affects immune cells that contribute to the microenvironment and impact cancer progression. The impact on tumor initiation ageing also requires an age-stratified analysis of treatment responses, especially immunotherapy in oncology (Dolan et al., 2025).

Interestingly, the immune system of the aging organism resembles that of the newborn, presenting Neu and Mf with reduced antimicrobial activity, DC with reduced Ag-presenting function, impairment of NK cells, and compromised adaptive lymphocyte responses. The most critical change in the aging innate immune system is the increased production of proinflammatory cytokines IL-6, IL-1β, IL-15, and IL-18 and tumor necrosis factor (TNF)-α, the inflammation likely contributing to age-related diseases, including cancer.

Innate immune system cells contribute to the process of inflammaging by producing cytokines associated with chronic inflammation, while important cellular functions such as phagocytosis, Ag uptake and presentation, cellular migration, and bactericidal activity decline with aging (Batista et al., 2020).

Interest in inflammaging has been spurred by recent research demonstrating roles for senescent cells in driving chronic inflammatory signaling and defining the cellular and molecular triggers that sustain cytokine production during aging. Alongside increases in pro-inflammatory mediators (e.g., IL-6, TNF-α, and IL-1β), attention to anti-inflammatory mediators (e.g., IL-10 and IL-1Ra) and composite ratios (e.g., IL-6: IL-10) can better index inflammatory balance in older adults. Chronic inflammation remodels the elements of the immune system, with overall consequences upon the health of elderly (Karpuzoglu et al., 2025).

Subtle mechanisms such as cellular senescence, mitochondrial dysfunction, defective autophagy, inflammasome activation, activation of the DNA damage response, and telomerase shortening favor deregulation in microbiome composition and in chronic cytomegalovirus infections (Fulop et al., 2023). Cellular degradation, including lysosomal (autophagy) and proteosomal degradation, as well as protein catabolism supported by cytosolic and mitochondrial proteases, induces the continuous turnover of damaged cellular components. Incomplete and/or inefficient degradation processes induce the progressive accumulation of biological “garbage”, such as oxidized proteins, dysfunctional mitochondria, and biological molecules that are resistant to intra-lysosomal degradation. Autophagy is a complex proteolytic pathway that transfers aged cellular components to the lysosomal compartment for their degradation. ROS production has been shown to be reduced in autophagy-deficient neutrophils, and the inhibition of nicotinamide adenine dinucleotide phosphate (NADPH) oxidase decreases Neu degranulation. There is a balance between these molecules, with ROS generation inducing autophagy—a process necessary to maintain efficient ROS production (Kma and Baruah, 2022). Aging is a complex process based mainly on ROS-induced cellular damage. In the mitochondria of aerobic organisms, the electron transport chain plays a major role in cellular energy production and is a significant source of ROS that damage DNA, RNA, and cellular proteins.

In the elderly, vulnerability to infection may be the consequence of a reduced number of innate immune cells reaching the inflammatory site or a decrease in intrinsic antibacterial activity; therefore, response to infection and vaccination decreases with age.

According to the World Health Organization (WHO), the global percentage of people over 60 years of age is increasing faster than for any other age group. In 2025 it reached 1.2 billion people, and by 2050 it will reach 2 billion, 80% of whom will live in developing countries. Recently, Eurostat Statistics estimated that the population of the European Union will grow 2.5 times between 2021 and 2100, from 6.0% to 14.6%, so therefore the elderly population will increase. As there is a rapid increase in the elderly population in developed countries, healthcare is faced with increased morbidity due to age-related diseases such as infections and cancers, and consequently with a declining response to vaccination. The reasons for these effects are complex, but the reduced efficiency of the innate and adaptive immune system is likely essential to the pathologies associated with advanced age.

However, a remarkable heterogeneity in aging trajectories has been noted, with a significant proportion of individuals undergoing an adaptive remodeling and maintenance of their immune equilibrium in spite of their advanced age. This group encompass centenarians, who exhibit low levels of chronic inflammation, robust responses against infectious challenges, and reduced susceptibility to age-related disorders alongside extended lifespans and physical independence. They have a unique immune cell composition and activity, characterized by the predominance of cytotoxic cell lineages such as T-CD4^+^ and T-CD8^+^ cells, retention of naïve T cells, and preservation of Neu and NK cells functions, which contributes to stress adaptation and limited inflammation (Anaya et al., 2026). Another distinctive feature of centenarians and supercentenarians is the abundance of peripheral differentiated B cells (which produce high-affinity Abs) and an enhanced antigen presentation of Mo CD14^+^, emphasizing sustained immune activity. This contrasts with increased abundances of naïve T cells and Tregs observed in elderly controls, suggesting potential biomarkers for longevity assessment in clinical cohorts (Wang Y. et al., 2025). All these changes observed in adults and centenarians are summarized in Figure 4.

**FIGURE 4:**
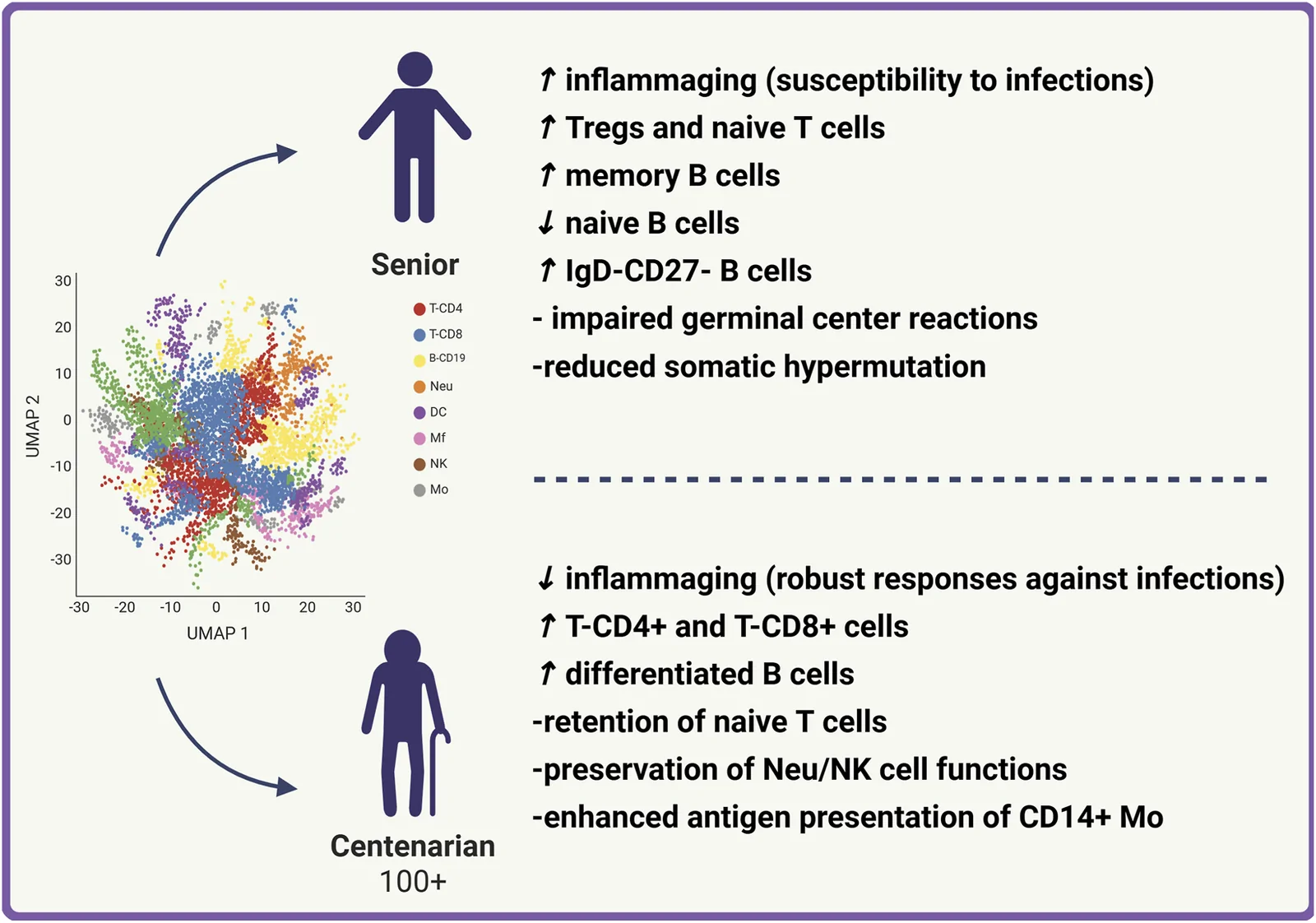
Different trajectories of age-related changes in the elderly. Comparative evaluation of immune cell populations between seniors and centenarians. Created in https://BioRender.com.

Therefore, although the mechanisms responsible for these age-related changes in innate and adaptive immunity are not fully understood, recent progress in high throughput omics technologies promises better strategies to overcome them in the future (Batista et al., 2020).

## 6 Conclusion

Both innate and adaptive immune systems in newborns and the elderly are deficient, making them highly susceptible to infections and thus contributing to increased mortality and morbidity. All defense mechanisms change in order to adapt, first to fetal–infant changes, then the process continues with maturation and expansion during growth. It is not immaturity but a highly regulated tolerogenic status that distinguishes the neonatal innate immune system from that of adults. Neonates are immunologically challenged directly after birth by a rigorous change from a semi-allogeneic sterile environment into a world rich with microbes. A so-called disease tolerogenic state is typical for neonates and is anticipated to prevent immunopathological damage, potentially at the cost of uncontrolled pathogen proliferation. Consequently, neonates are more susceptible than adults to life-threatening infections, and this trend becomes more evident in pre-term newborns, who have inferior immune functions and higher mortality and morbidity rates. Thus, understanding the characteristics of human early-life immunity is crucial to the design of immune-based interventions, including the development of vaccines strategies, that may promote pediatric health. During lifetime, the immune system undergoes a profound remodeling, and with ageing, immune senescence occurs, impacting overall health and survival.

Aging can follow diverse trajectories; however, centenarians, in particular, may offer valuable insights into the immune mechanisms and biomarkers associated with longevity and the mitigation of inflammaging phenomenon. The study of longevity-related immune mechanisms is a nascent yet highly dynamic field that could soon lead to the development of novel therapies to prevent age-related disorders in adults and restore immune cell function in frail individuals. Given the global phenomenon of demographic aging alongside its associated morbidity, mortality, and socioeconomic costs, an expansion of research in this field is urgently needed to overcome the geriatric burden worldwide.
